# Credential farming in the age of generative AI: relocating trust from outputs to communities

**DOI:** 10.21203/rs.3.rs-10944586/v1

**Published:** 2026-10-08

**Authors:** Gift Osei Agyemang, Rawad AL-Louzi, Melat Mezgebu Bawoke, Connor Bender, Leo Anthony Celi, Nishita Chavan, Rahul Gorijavolu, Diego Halac, Nikhil Jaiswal, Sofiya Mikolutskaya, Anthony Nguyen, Raul Ignacio Rojas Sillery, Sparsh Roy, Dharaa Suresha, Alexa Xiang, Boya Zhang

**Affiliations:** University of Energy and Natural Resources; Al-Balqa Applied University; Addis Ababa University; Hackensack Meridian School of Medicine; Massachusetts Institute of Technology; East Brunswick High School; Johns Hopkins University; Sanatorio Anchorena del Callao; Massachusetts Institute of Technology; Troy High School; Troy High School; University of Florida; Hopewell Valley Central High School; Dwight Global Online School; The Ohio State University; Massachusetts Institute of Technology

## Abstract

Publications, affiliations and authorship positions began as proxies for qualities that cannot be observed directly: judgement, understanding and the capacity to contribute to shared inquiry. Generative artificial intelligence has weakened the inference those proxies depend on. Polished research outputs can now be produced by people who do not understand them, yet admissions committees and hiring panels still read an output as evidence of the competence that producing it once required. The result is faster credential farming, the extraction of the social trust held in credentials without contribution to the enterprise that gives them meaning. Better detection of machine-generated text will not repair this. What has to change is where epistemic trust sits. Work in the sociology of credentials, in social epistemology and on situated learning points to where it should move: scientific formation, how communities shape people and teach them what to value, carries the evidential weight that credentials only summarised. Three commitments follow. Select members for depth rather than legibility. Build small relational structures in which judgement can be formed and witnessed. Refuse to convert those structures into new metrics. The argument matters most for researchers in low-resource settings, whose learning happens in informal spaces that credentialing systems do not see, and who are asked to make themselves legible in a vocabulary nobody taught them.

## Introduction

A secondary-school student can now co-author a polished preprint, list a laboratory affiliation and present to an admissions committee as a scientific contributor, without having learned much in the process. Little in the resulting portfolio separates that student from a peer who spent two years earning the same lines. The problem is evidential. The documents that institutions use to infer competence no longer support that inference reliably.

Credentials began as proxies for qualities nobody can observe directly (Spence, 1973), and generative artificial intelligence has loosened the link between producing a research artefact and possessing them. An arms race of detection will not restore it. Trust has to move off outputs, which can be manufactured, and onto formation inside research communities, which is harder to fake. Selection and mentorship should be rebuilt on that basis.

This argument comes out of discussions within MIT Critical Data, a global open-science community built around a widely used critical care database (Johnson et al., 2016). It does not speak for any institution. We write from inside the tension we are describing: communities that benefit from the symbolic power of elite affiliations attract, by that same power, people who want the symbol more than the work.

## Credentials were always proxies

A credential is compressed testimony. A doctorate certified that its holder had survived years of supervised apprenticeship; a first-authored paper certified that its author did the intellectual work the byline implied. Collins (1979) argued that credentials did something else at the same time: they rationed access to status and worked as currency in a stratification system only loosely coupled to competence. The two functions coexisted for most of the twentieth century, because producing the artefact required the competence.

What happens to proxies under pressure is well documented. High-stakes quantitative indicators corrupt the processes they are meant to track (Campbell, 1979), and, in Strathern’s (1997) formulation of Goodhart’s (1975) law, a measure that becomes a target ceases to be a good measure. Academic science has supplied three decades of illustration. The h-index and the journal impact factor became units of professional advancement, and researchers learned to optimise the unit rather than the understanding it once indicated (Muller, 2018). The research-assessment community has conceded the point in principle: both the Leiden Manifesto and the San Francisco Declaration on Research Assessment tell evaluators to stop outsourcing judgement to metrics (DORA, 2013; Hicks et al., 2015). The metrics persist anyway, because they still predict who gets scarce positions even as their epistemic meaning drains away.

The motivational residue of all that optimising matters here. Extrinsic rewards can crowd out intrinsic interest (Deci and Ryan, 1985), and intrinsic and prosocial motives are associated with more creative and more useful work (Grant and Berry, 2011). Merton (1973) counted communism, the shared ownership of findings, among the norms of science; an enterprise built on sharing depends on members who are motivated to contribute rather than to extract. Credential farming, our term for taking the social trust held in credentials without contributing to the enterprise that gives them meaning, is therefore more than a fairness problem. Over time it changes what science gets done.

Figure 1 sets the two dynamics side by side. The difference is structural. Credential farming closes into a loop, in which the weakened signal becomes the reason to produce more output, so the cycle accelerates as its evidential value falls. Formation does not close. It accumulates, and each stage leaves behind someone who can vouch for what happened.

## What generative AI changed

Every credential system runs on one inference: the artefact exists, so its author has the competence that producing it requires. Large language models have weakened that inference. Polished prose, literature syntheses, working code and plausible analyses are now available to people who understand little of it (Kasneci et al., 2023). Much of the education literature treats this as an integrity crisis to be met with detection (e.g. Cotton et al., 2024). That framing is too small. Detection tools are unreliable and biased against writers whose first language is not English (Liang et al., 2023; Weber-Wulff et al., 2023), and they defend a boundary that has stopped marking much. The damage is to validity: the artefact was evidence of competence because of what producing it demanded, and it demands less now (Dawson et al., 2024).

A fast-growing literature makes a related diagnosis. Signalling accounts hold that generative AI compresses the differential cost of producing academic outputs and so erodes their credibility as evidence of competence (Tamir and Nairz-Wirth, 2026). Work on assessment argues that the assumption linking the quality of a written product to the ability of its producer has quietly collapsed (Inoshita et al., 2026). Interview studies of doctoral students describe scholarly labour shifting from production towards verification (Wu, 2026). These accounts locate the remedy in verification: better checks, oral examination, proof of process. We accept the diagnosis and press the remedy further. Verification still treats the individual artefact as the unit of evidence and asks only that it be checked more carefully. Our claim is that the evidential weight was never in the artefact. It lies in formation, in the sustained and witnessed participation through which a community comes to know what someone can do, and evaluators should look there rather than at outputs. That claim also reaches researchers formed in low-resource settings, whose formation happens in spaces institutions do not see and whom the verification literature has not addressed.

Three qualifications guard against overstating this. AI has not made knowledge worthless. It has made unwitnessed output weaker evidence of judgement and responsibility. Someone who uses a model as an instrument of thinking may learn more and faster, and the difficulty is that their artefacts look much like those of someone who learned nothing. The inference was also fraying well before 2022, as paper mills and honorary bylines diluted it. Generative AI did not invent the split between production and comprehension. It made the split cheap and available to anyone who can write a prompt. Nor has the inference collapsed everywhere at once. An audit of the largest data-science competition platform across the AI transition found that freshly earned credentials kept most of their predictive value, while stocks of older credentials from assessments that had stopped running lost most of theirs (Yao, 2026). Credentials, on that evidence, are only as informative as the institutions that keep renewing them, which is our point: their value was never stored in the artefact but in an ongoing practice of assessment that someone has to maintain.

Evidential weight has to go somewhere. If artefacts can be manufactured, whatever is harder to manufacture appreciates: participation sustained and witnessed over time, positions defended and revised in dialogue, errors owned in front of people who can tell the difference. Communities observe this. Credentials compress it away.

The cost of leaving this alone is already visible. Admissions committees and hiring panels continue to ask for more artefacts, earlier. More preprints from teenagers (Golden and Purohit, 2023), more repositories, more performance. Each increment selects harder for access and coaching and more weakly for understanding. If the race between manufactured signals and signal-hungry institutions continues, credentials will tell evaluators progressively less about competence.

## A problem of formation

When trust in science erodes, the reflex response is informational: more transparency, better communication. We read the problem instead as one of formation, of how research communities shape people. Several literatures support that reading.

Biesta (2009) separates three functions of education: qualification, socialisation and subjectification. Credentialing systems measure the first, and the other two wither for want of indicators. Socialisation into a practice, and the formation of people who care about truth, are what scientific communities run on. Longino (1990, 2002) gives the epistemological version: scientific knowledge is social, produced through criticism, public standards and what she later called a tempered equality of intellectual authority. Honesty and openness to criticism are social virtues. They can only be learned in relation to others.

Situated learning theory describes the mechanism. Lave and Wenger (1991) treat learning as a trajectory of legitimate peripheral participation rather than a transfer of information into individual heads. Newcomers become knowers by doing progressively more consequential work inside a community of practice, in view of people who can recognise quality. Polanyi (1966) supplies the reason codified knowledge cannot stand in for this: we can know more than we can tell. A learner can acquire the definition of confounding without acquiring any sense of when it matters, or when a technically correct answer should still produce doubt. That judgement passes through apprenticeship, by watching another person work with their mistakes and revisions visible. It is also what survives generative AI, because it never lived in artefacts.

We are not proposing that communities replace credentials. Credentials were always an IOU written against community formation, and AI has forced the question of whether the underlying asset exists. Where competence has been formed and witnessed in relationship, a community can testify to it directly. Where it has not, the credential certifies much less than it appears to.

## What research communities can do

Moving trust from outputs to formation requires structures in which formation can be seen. Our community is piloting three. They are design principles to test, and we have no evidence yet that they work.

## Select for depth rather than legibility

Credentials survive in selection because they are auditable and character is not. Live, unscripted conversation recovers some of that observability. We have moved towards open discussions with prospective members built around questions that have no correct answers. When did something last unsettle you enough to change how you saw the world? What would you do on finding a significant error in a paper you had co-authored? A language model can rehearse an answer to these. What it cannot supply is the thinking behind one: the hesitation and the revision that separate a position held from a position performed. The questions also do formative work whatever the outcome, since they show a candidate the culture they would be entering. Assessment of this kind is subjective, and we treat it as one weak signal among several. A single conversation would reproduce the problem we describe below, since it rewards those already fluent in narrating themselves. So it is not where the decision is made. Entry is provisional, and what counts is what a person shows over the months that follow: whether they ask questions, own errors and change their mind in front of others. The conversation begins that record. It does not stand in for it.

## Build small relational infrastructure and keep it unmeasured

Peer-to-peer learning yields cognitive gains for tutor and tutee alike (Topping, 1996), and disagreement surfaces more readily where psychological safety exists (Edmondson, 1999). Between large project meetings we assign groups of two or three members with no faculty present, whose task is to continue the conversation rather than to produce anything. What did I understand this week that I did not understand before? When did I change my mind? Low-friction messaging channels give these exchanges continuity and recover something of the corridor conversation that formal structures squeeze out. Versions of this already exist in low-resource settings, where formal mentorship is thin and necessity built them. A case-discussion group running on a cheap messaging platform creates the same safety to say “I do not know”. Global research communities should connect to those spaces rather than duplicate them.

## Refuse to metricise the alternative

Measurement pulls hard. Left alone it will convert participation into attendance counts and response times, and rebuild the credential-farming machine inside the reform (Campbell, 1979). The distinction that matters is between measurement and judgement. A measure is quantified, comparable across people and therefore optimisable. A judgement is qualitative, tied to its context and made by someone who can be asked to defend it. Formation can be judged, by asking over time whether members change their minds and whether intellectual relationships outlast the tasks that created them, but the moment those judgements are scored and ranked they become targets. Judgement has to stay subordinate to the thing it tracks, or measurement will replace both.

## The formation gap

The stakes are unevenly distributed. In many education systems, academic selection remains strictly grade-based. Holistic assessment, exported as the meritocratic alternative, carries a hidden curriculum: it rewards people trained to present themselves as curious and community-minded, and that training is unequally distributed. A researcher formed entirely within a grade-based system may have deep curiosity and real epistemic humility and still find the question “when did something last unsettle you?” unanswerable, because nobody taught them that such experiences count as evidence of anything. This is close to what Fricker (2007) calls hermeneutical injustice: a significant area of a person’s social experience obscured from collective understanding because structural prejudice has kept their group from shaping the shared interpretive resources. The parallel holds because the vocabulary of holistic assessment was built by and for the institutions that export it, not by those formed within grade-based systems, so the interpretive gap falls on the latter and stops them rendering their own experience legible to institutions with power over them.

Informal study groups and peer networks are where many researchers in low-resource settings form one another. That learning is real and institutionally invisible, the inverse of credential farming, in which the institutionally visible turns out to be epistemically empty. A community serious about recruiting beyond its existing networks has to do more than find these researchers. It has to create conditions where relational and practical intelligence becomes visible on its own terms, without fluency in a self-presentation vocabulary nobody taught them.

## Limits and risks

We have not solved credential farming, character is not reliably observable, and our selection practices are not demonstrably fair. Nor do we blame individuals for structural incentives. A student who needs elite placement to secure their family’s financial stability is not making a mistake by optimising for credentials, and telling young people to stop caring about prestige without changing what prestige buys is neither kind nor realistic. Selection built on conversation carries its own bias, against candidates from places where self-narration is not a trained skill. Communities with strong shared values carry their own pathologies: insularity, and the ease with which “we select for depth” becomes a gatekeeping mechanism much like the prestige system it replaced. We are building in accountability through ethics oversight of any formal study of our community, documented decision-making and rotating leadership. Four of the authors are secondary-school students, two of whom led the conceptualisation and drafting of this Comment, and the profile sketched in its opening sentence is our own. We cannot certify our own formation from the inside, and readers should discount our self-report accordingly. The argument does not require us to be exceptions to it. It predicts that people in our position will be hard to tell apart from those who learned nothing, which is precisely the discrimination we are asking research communities to make. A peer-reviewed critique of academic performance is itself an academic performance, and we accept that tension, since institutional change is usually made by people working inside the structures they want to outgrow.

## Conclusion

A practical agenda follows from the argument. Research communities should pilot selection and mentorship structures that make formation observable, and document them honestly, failures included. Funders and institutions that signed the research-assessment reforms should extend them past outputs to evidence of formation: testimony to witnessed judgement, participation that peers will vouch for. Educators should treat generative AI less as a cheating problem to be policed and more as the end of artefact-based inference, and rebuild assessment around process and revision (Swiecki et al., 2022). Global research networks should find the informal learning communities that already exist in low-resource settings and connect to them as peers.

A community offers something a credential cannot: a conversation that goes somewhere neither person expected, after which both know something they did not know before. No institution can mass-produce that. Institutions can decide whether their incentives make it more or less likely, and for most of a century they could avoid deciding, because the credential system summarised formation well enough. Generative AI has made that option harder to hold. Machines can write the papers; they cannot become someone worth trusting. Telling the difference is now a central task for scientific institutions.

## Figures and Tables

**Figure 1:**
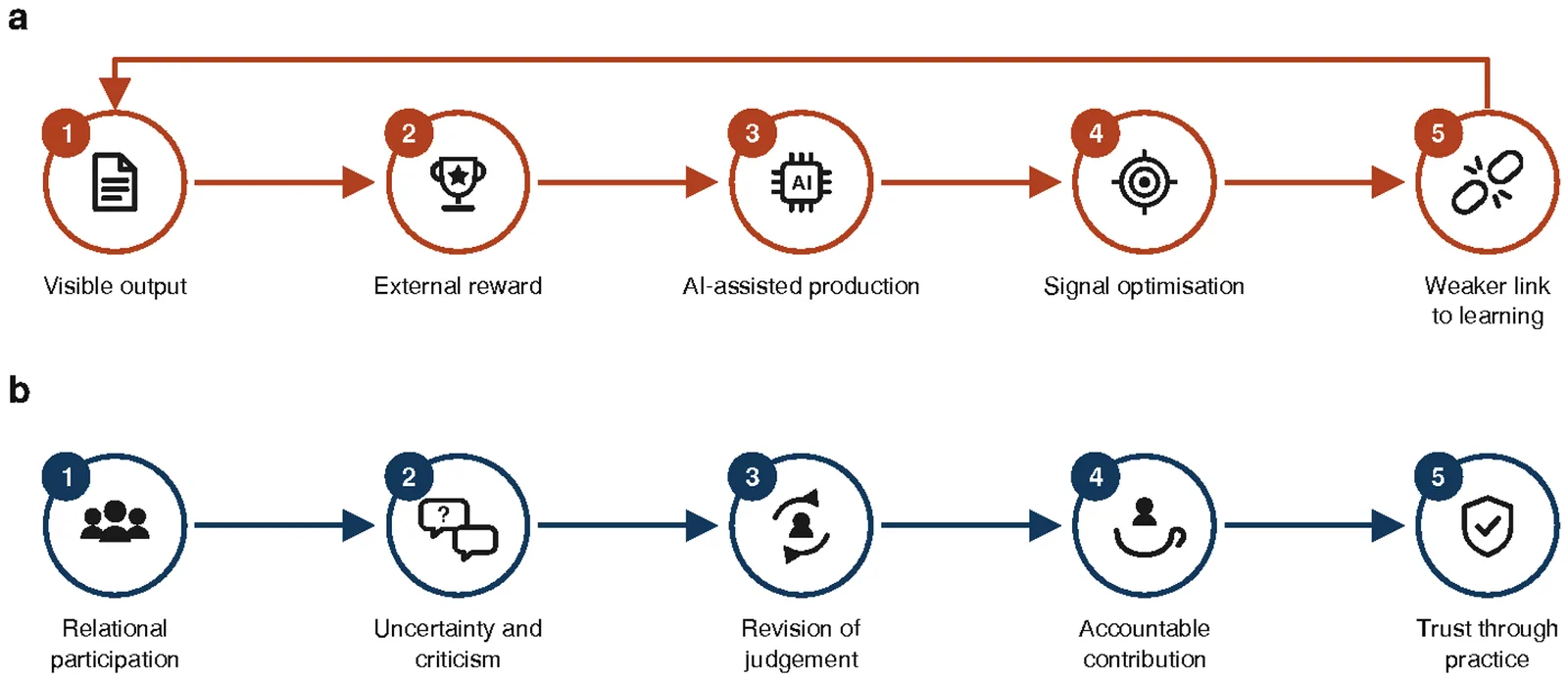
Two ways trust can be built in a research community. **a** The credential-farming cycle, in which visible output earns external reward, AI-assisted production lowers the cost of that output, effort shifts to optimising the signal, and the link between the signal and any underlying learning weakens; the weakened signal feeds back into further output. **b** Formation through practice, in which relational participation makes uncertainty and criticism expressible, judgement is revised in front of others, contribution becomes accountable, and trust accumulates through witnessed practice rather than through artefacts.

## Data Availability

Data sharing is not applicable to this article as no datasets were generated or analysed during the current study.
